# Activation of cannabinoid receptor type II by AM1241 protects adipose-derived mesenchymal stem cells from oxidative damage and enhances their therapeutic efficacy in myocardial infarction mice *via* Stat3 activation

**DOI:** 10.18632/oncotarget.17614

**Published:** 2017-05-04

**Authors:** Dong Han, Xiang Li, Wen-Si Fan, Jiang-Wei Chen, Tian-Tian Gou, Tao Su, Miao-Miao Fan, Meng-Qi Xu, Ya-Bin Wang, Sai Ma, Ya Qiu, Feng Cao

**Affiliations:** ^1^ Department of Cardiology, State Key Laboratory of Kidney Diseases, Chinese PLA General Hospital, Beijing, China; ^2^ Department of Cardiology, Xijing Hospital, Fourth Military Medical University, Xi’an, Shaanxi, China

**Keywords:** cannabinoid receptor type II, stem cells, oxidative stress, myocardial infarction, Stat3, Pathology Section

## Abstract

The poor survival of cells in ischemic sites diminishes the therapeutic efficacy of stem cell therapy. Previously we and others have reported that Cannabinoid receptor type II (CB2) is protective during heart ischemic injury for its anti-oxidative activity. However, whether CB2 activation could improve the survival and therapeutic efficacy of stem cells in ischemic myocardium and the underlying mechanisms remain elusive. Here, we showed evidence that CB2 agonist AM1241 treatment could improve the functional survival of adipose-derived mesenchymal stem cells (AD-MSCs) *in vitro* as well as *in vivo*. Moreover, AD-MSCs adjuvant with AM1241 improved cardiac function, and inhibited cardiac oxidative stress, apoptosis and fibrosis. To unveil possible mechanisms, AD-MSCs were exposed to hydrogen peroxide/serum deprivation to simulate the ischemic environment in myocardium. Results delineated that AM1241 blocked the apoptosis, oxidative damage and promoted the paracrine effects of AD-MSCs. Mechanistically, AM1241 activated signal transducers and activators of transcription 3 (Stat3) through the phosphorylation of Akt and ERK1/2. Moreover, the administration of AM630, LY294002, U0126 and AG490 (inhibitors for CB2, Akt, ERK1/2 and Stat3, respectively) could abolish the beneficial actions of AM1241. Our result support the promise of CB2 activation as an effective strategy to optimize stem cell-based therapy possibly through Stat3 activation.

## INTRODUCTION

Coronary heart disease (CHD) is the leading cause of morbidity and mortality worldwide. In terms of current therapeutic options for CHD, stem cell-based therapies hold some great promises for heart regeneration [1]. Nevertheless, the therapeutic efficacy has been hindered by the poor survival of engrafted cells. Within the first few days after transplantation, the survival and retention of MSCs is largely endangered by the harmful microenvironment of ischemia [2]. Myocardial oxidative stress, and other pro-apoptotic factors severely restrain the therapeutic effects of MSCs on cardiac repair [3]. To this end, it is reasonable to search for novel strategy to promote donor cell survival for the purpose of achieving the optimal efficacy of stem cell-based therapy for CHD.

The cannabinoid receptor-type II (CB2 receptor) is a widespread seven-transmembrane and G protein-coupled receptor in various cells and tissues including liver, skin, heart, immune cells, mesenchymal stem cells, etc [4, 5]. In the past several years, the CB2 receptor activation has emerged as a promising therapeutic target to protect against cardiac and cerebral ischemic diseases. Our previous studies also suggested that CB2 receptor agonist AM1241 activated PI3K/Akt/Nrf2 signaling to reduce excess oxidative stress, thereby promoting endogenous myocardial regeneration and alleviating myocardial fibrosis in ischemic heart [6, 7]. On the other hand, CB2 activation has been closely associated with mesenchymal stem cell behaviors. CB2 was revealed to be a crucial mediator of mesenchymal stem cell immunosuppressive properties, migration, proliferation and differentiation [8-11]. However, studies addressing the impact of CB2 activation on survival and therapeutic efficacy of mesenchymal stem cells in ischemic myocardium and the underlying mechanisms are still lacking.

Signal transducers and activators of transcription 3(Stat3) signaling, a highly evolutionarily conserved pathway, has been revealed to be responsible for the cardio-protective effects of various interventions, including pharmacological and non-pharmacological treatment of myocardial ischemic injury [12]. Notably, activation of Stat3 could improve the ischemic cardiac microenvironment by fine-tuning myocardial processes such as apoptosis, autophagy, inflammation, and oxidative stress. Furthermore, activation of Stat3 has been proved to protect mesenchymal stem cells from oxidative damage and enhances their therapeutic potency in infarcted myocardium [13].

This study therefore aimed at evaluating the hypothesis that activation of CB2 protects mesenchymal stem cells from oxidative damage and enhances their therapeutic potency in infarcted myocardium through Stat3 signaling pathway.

## RESULTS

### CB2 agonist promoted survival of engrafted AD-MSCs in ischemic myocardium

**Figure 1:**
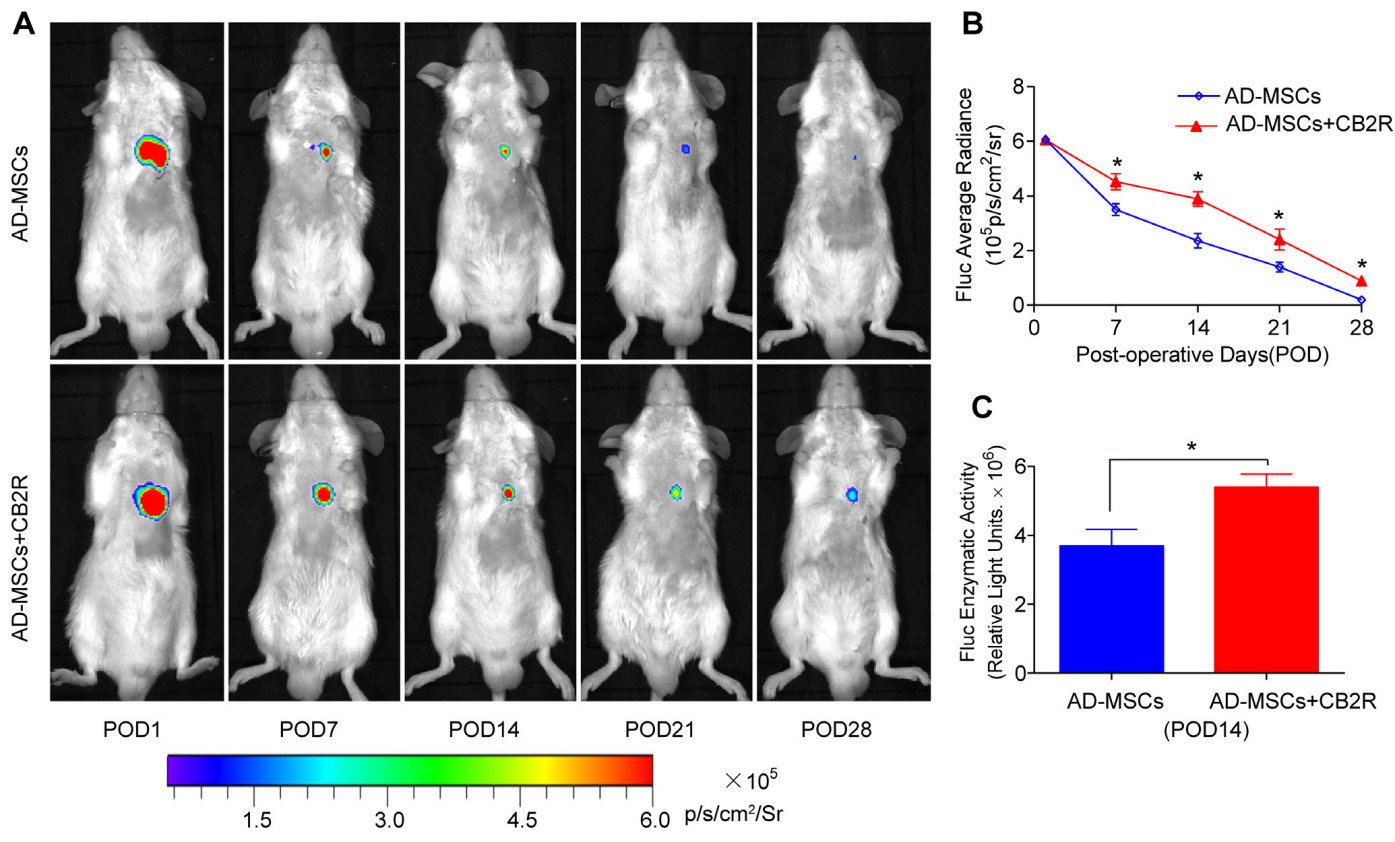
CB2 agonist promoted survival of engrafted AD-MSCs in ischemic myocardium **A.** Longitudinal BLI tracked the survival and retention of AD-MSCs survival *in vivo* (*n* = 8 for each group). Colored scale bar represents BLI radiance intensity in p/s/cm ^2^/sr. **B.** Quantitative analysis of A. **C.** *Ex vivo* Fluc enzymatic activity of infarcted myocardium on POD 14 (*n* = 5). . *P < 0.05 *versus* AD-MSCs.

Our previous studies have demonstrated that bioluminescence imaging was a reliable tool to monitor the fate of AD-MSCs transplanted into infarcted myocardium. As shown in Figure 1A, 1B, BLI signal did not differ among groups on POD1 (*P* > 0.05). However, on postoperative day (POD) 7, 14, 21 and 28, the BLI signals in CB2 agonist treated group were significantly higher than that in untreated group(*P* < 0.05). This was also confirmed by the significantly higher Fluc enzymatic activity in the CB2 agonist treated group on POD14 (*versus* the AD-MSCs group, *P* < 0.05, Figure 1C). These results illustrated that CB2 agonist treatment could significantly improve the retention and survival of engrafted AD-MSCs in ischemic myocardium.

### CB2 agonist combined AD-MSCs significantly improved cardiac function after MI

**Figure 2:**
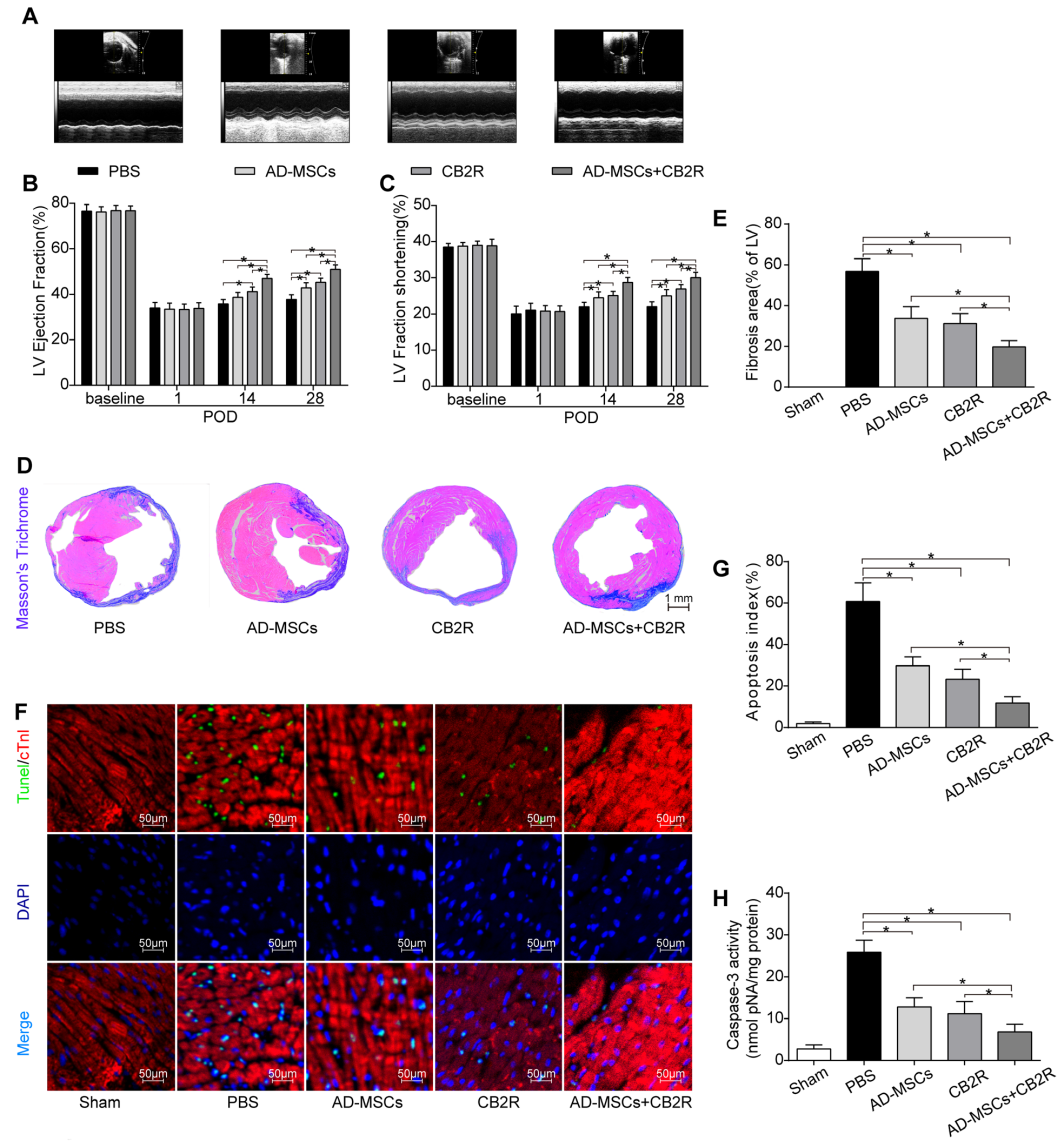
Effects of AD-MSCs and CB2 agonist treatment on post-MI cardiac function, cardiac fibrosis and apoptosis **A.** Representative M-mode images by echocardiography on POD28. Left ventricle ejection fraction **B.** and fractional shortening **C.** were calculated by M-mode echocardiography on baseline, POD 1, 14, and 28(*n* = 6). *P < 0.05 between indicated groups. **D.**, **E.** Myocardial fibrosis was determined by Masson’s trichrome staining (*n* = 6-8, bar = 1 mm). **F.** Representative TUNEL graphs depicting myocardial apoptosis on POD3. TUNEL-positive cells (green fluorescence), cTnI (red fluorescence), and DAPI-positive nuclei (blue fluorescence), bar = 50μm; **G.** Graphs summarize apoptosis index calculated by the number of TUNEL-positive nuclei per 100 nuclei from five randomly selected fields and **H.** Myocardial caspase-3 activity (*n* = 6-8) was measured. *P < 0.05 between indicated groups.

Serial echocardiographic analysis indicated that there was no significant difference in left ventricular ejection fraction (EF) and fraction shortening (FS) between all groups at baseline and POD1 (*p* > 0.05). On POD 14 and 28, EF and FS experienced a significant improvement in AD-MSCs, CB2R and AD-MSCs+CB2R group compared with PBS group, with the best parameters in AD-MSCs+CB2R group (Figure 2B, 2C; *P* < 0.05). A synergism was witnessed in combination with AD-MSCs and CB2 agonist treatment, which markedly improved the left ventricular parameters.

### CB2 agonist combined AD-MSCs treatment reduced myocardial fibrosis and apoptosis

We next sought to investigate the effects of CB2 agonist treatment on myocardial fibrosis and apoptosis. Masson’s Trichrome staining showed a marked reduction in left ventricle fibrosis area in AD-MSCs, CB2R and AD-MSCs+CB2R group compared with PBS group (Figure 2D, 2E; *P* < 0.05, respectively), with the least LV fibrosis in AD-MSCs+CB2R group (Figure 2D, 2E; *P* < 0.05). 3 days post operation, TUNEL assay was used to assess the level of apoptosis of cardiac cells in infarcted myocardium. Myocardial apoptotic index was significantly reduced in AD-MSCs, CB2R and AD-MSCs+CB2R group compared with PBS group (Figure 2F, 2G; *P* < 0.05, respectively), with the apoptotic index in AD-MSCs+CB2R group being the least (Figure 2F, 2G; *P* < 0.05). This result was confirmed by caspase3 activity in cardiac tissues(Figure 2H).

### CB2 agonist adjuvant with AD-MSCs leads to attenuation of myocardial oxidative stress

**Figure 3:**
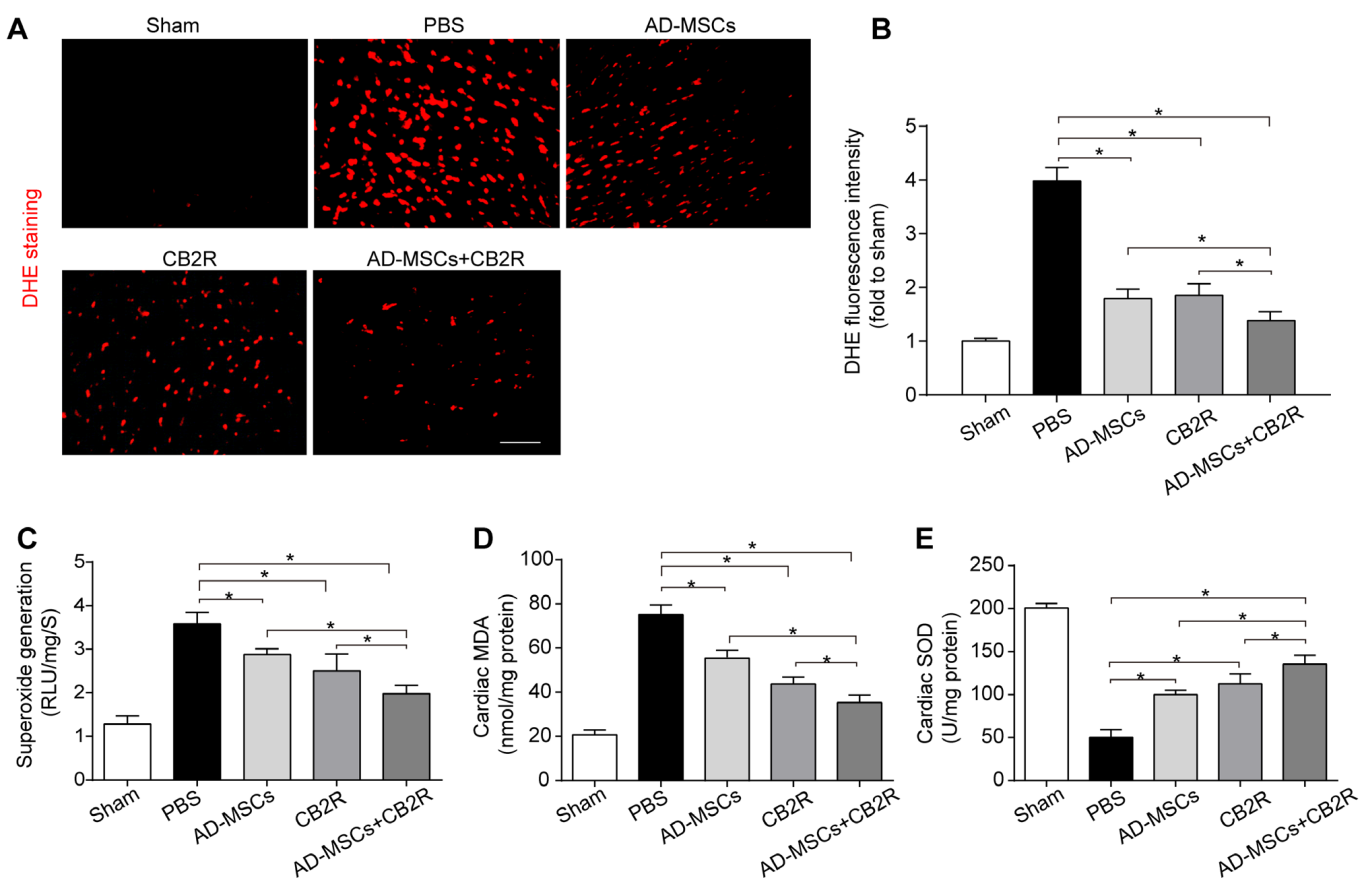
CB2 agonist adjuvant with AD-MSCs leads to attenuation of myocardial oxidative stress **A.**, **B.** Representative images of dihydroethidium fluorescence staining that evaluated ROS generation in myocardium and bar graph summarizing fluorescence intensity on POD3(*n* = 5). bar = 100μm; **C.** Myocardial levels of O_2_^-^ by lucigenin chemiluminescence method on POD3 (*n* = 5). **D.** Cardiac levels of MDA by enzyme-linked immunosorbent assay (ELISA) on POD3 (*n* = 5). **E.** Myocardial levels of SOD by testing kits. *P < 0.05 between indicated groups.

Overproduction of reactive oxygen species (ROS) is a critical feature of infarcted myocardium and contributes to cardiac injury [14]. We quantified myocardial O _2_^-^ content using both lucigenin-enhanced luminescence and dihydroethidium staining. Either AD-MSCs or CB2 agonist alone significantly lowered the MI-induced increase of O _2_^-^ generation(*versus* the PBS group, *P* < 0.05, Figure 3A, 3B, 3C), while combined treatment of AD-MSCs and CB2 agonist further decsssreased O 2- generation (*P* < 0.05, Figure 3A, 3B, 3C). Moreover, malondialdehyde (MDA, end-product of lipid peroxidation by reactive oxygen species) levels exhibited similar pattern as O _2_^-^ content (Figure 3D). In addition, SOD, a crucial myocardial endogenous antioxidant machinery, was also enhanced by either AD-MSCs or CB2 agonist treatment (*versus* the PBS group, *P* < 0.05, Figure 3E), with the most prominent enhancement in AD-MSCs+CB2R group (*P* < 0.05, Figure 3E).

### CB2 agonist adjuvant with AD-MSCs leads to myocardial activation of Stat3

**Figure 4:**
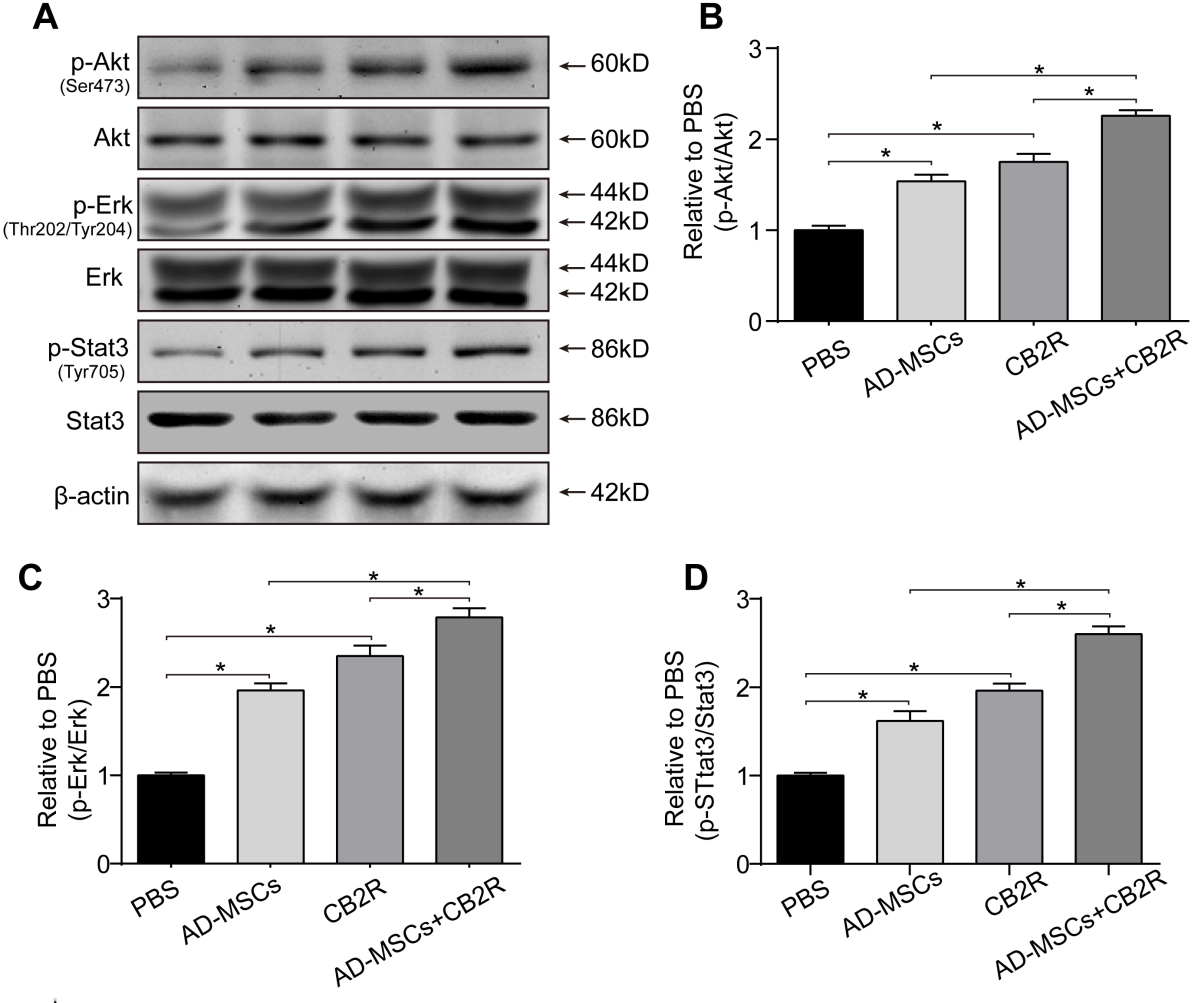
CB2 agonist adjuvant with AD-MSCs leads to myocardial activation of Stat3 **A.** Representative photographs of immune blot of Akt, Erk, Stat3 and their phosphorylation. (*n* = 3), **B.**-**D.** Quantitative analysis of **A.**. *P < 0.05 between indicated groups.

To gain insight into the mechanism involved in the protective effect of combined therapy, phosphorylation of Akt, Erk1/2 and Stat3 were examined by western blotting. Result showed that either CB2 agonist or AD-MSCs treatment significantly increased the levels of p-Akt(Ser473), p-Erk(Thr202/Tyr204)1/2, and p-Stat3 (Tyr705) (*versus* the PBS group, *P* < 0.05). Furthermore, combined treatment of AD-MSCs and CB2 agonist further strengthened this trend (*P* < 0.05, Figure 4A-4D).

### CB2 agonist attenuates H_2_O_2_ /SD-induced AD-MSCs injury and apoptosis

**Figure 5:**
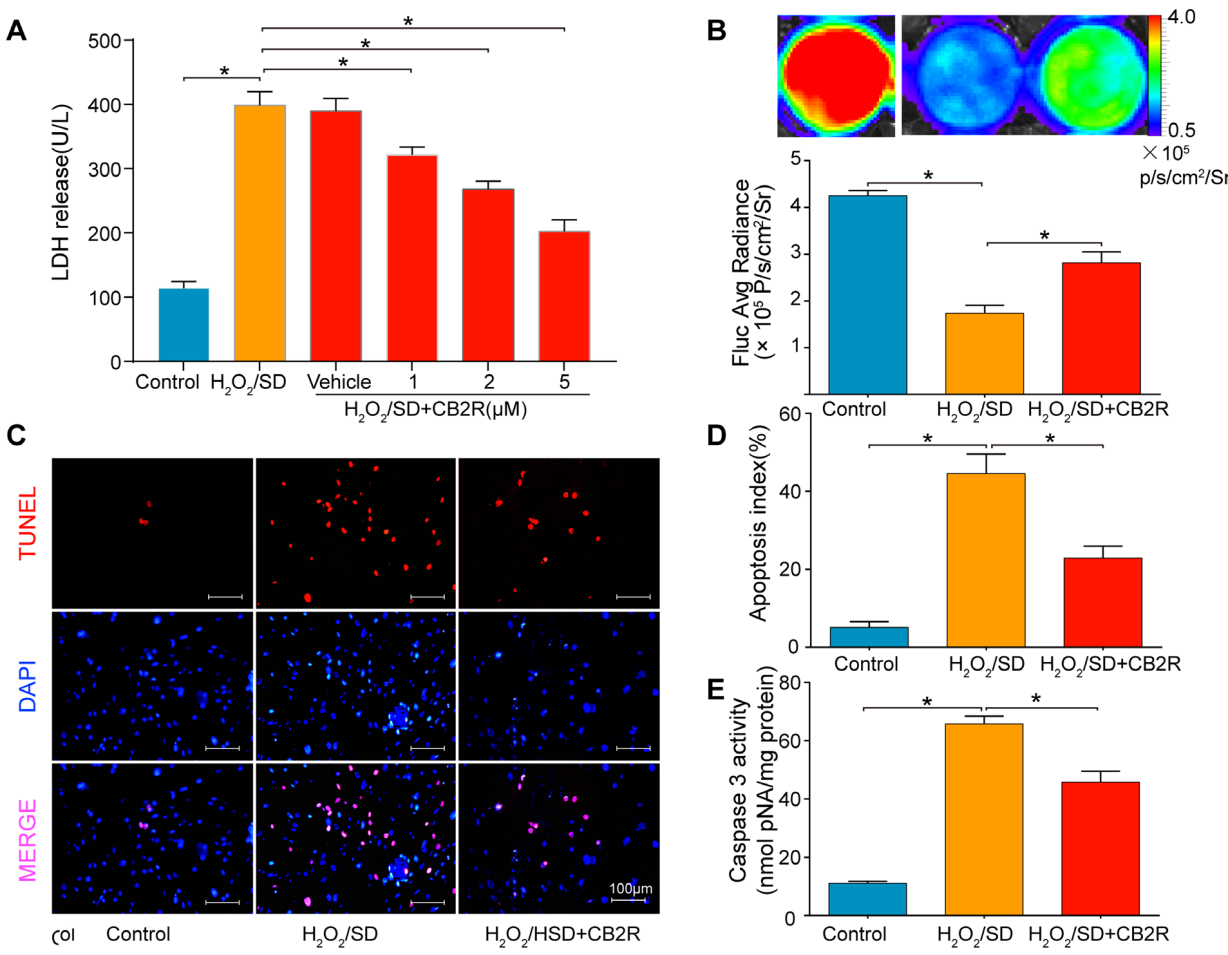
CB2 agonist attenuates H_2_O_2_/SD-induced AD-MSCs injury and apoptosis **A.** LDH release in AD-MSCs after respective treatment. **B.** *in vitro* BLI for AD-MSCs viability. **C.** Quantitative analysis of **B.**, *n* = 3. **C.** Representative photographs of TUNEL-stained AD-MSCs indicating cell apoptosis. **D.** Quantitative analysis of **C.**, *n* = 6. **E.** Casepase3 activity in AD-MSCs, *n* = 6. *P < 0.05 between indicated groups.

To reveal possible mechanisms, AD-MSCs were subjected to hydrogen peroxide/serum deprivation (H_2_O_2_/SD) injury to simulate ischemic and oxidative conditions *in vivo*. AD-MSCs were pre-treated with CB2 agonist AM1241 at concentrations of 1, 2, 5 μM for 12 hrs. Then they were exposed to 100μM H_2_O_2_/SD for another 12 hrs. As LDH was accepted as a sensitive index of MSCs damage, we examined LDH release in MSCs to evaluate the level of MSCs damage. The result showed that all three concentrations of CB2 agonist significantly inhibited H_2_O_2_ /SD-induced LDH release and the optimal effect was achieved by 5 μM (*P* < 0.05, Figure 5A). Further *in vitro* studies used 5μM as the optimal CB2 agonist dose.

*In vitro* BLI revealed that H_2_O_2_/SD injury for 12 hrs markedly decreased the viability of AD-MSCs while 5μM CB2 agonist significantly increased the viability of AD-MSCs (*versus* H_2_O_2_/SD group, *P* < 0.05, Figure 5B). Furthermore, TUNEL assay and measurement of caspase-3 activity were used to verify whether H_2_O_2_/SD induced AD-MSCs apoptosis could be reversed by CB2 agonist. Both apoptosis index(revealed by TUNEL assay) and caspase-3 activity were significantly inhibited by CB2 agonist(*P* < 0.05, Figure 5C-5E).

### CB2 agonist decreased oxidative stress levels in AD-MSCs subjected to H_2_O_2_/SD

**Figure 6:**
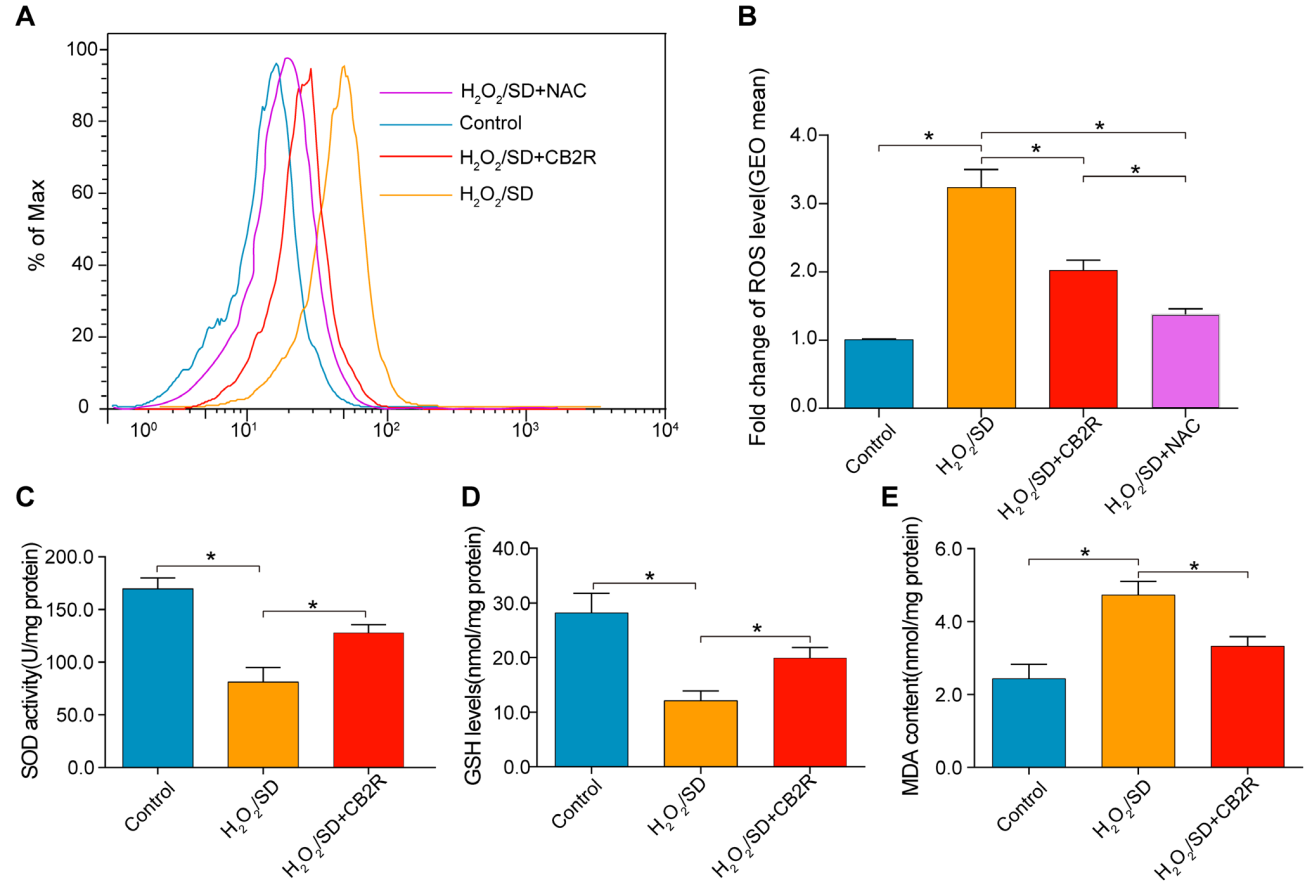
CB2 agonist decreased oxidative stress levels in AD-MSCs subjected to H2O2/SD **A.** Flow cytometry analysis of ROS production in AD-MSCs after indicated treatment, *n* = 3. **B.** Quantitative analysis of **A.**, **C.**-**E.** The SOD, GSH, and MDA level determined by respective commercially available assay kit. *n* = 3-6. *P < 0.05 between indicated groups.

**Figure 7:**
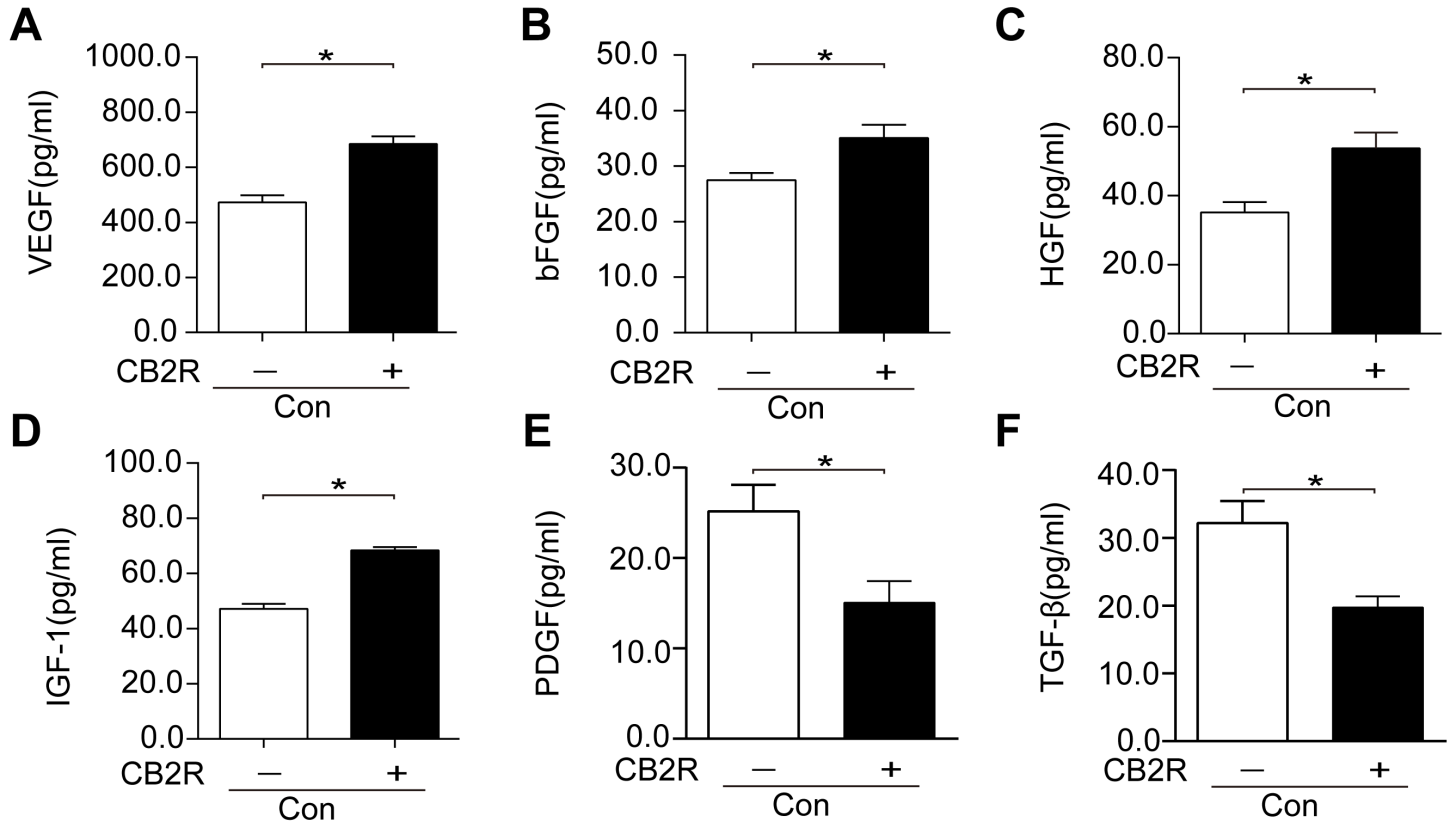
CB2 agonist enhanced the production of paracrine growth factors and inhibited profibrotic cytokines in AD-MSCs (**A-F**) Paracrine growth factors including VEGF, bFGF, HGF, and IGF-1 as well as profibrotic cytokines including PDGF andTGFβ were examined by respective ELISA kits. *P<0.05 between indicated groups..

ROS was detected by ROS probe DCFH-DA on a flow cytometer. As is shown in Figure 7, Fluorescence intensity enhanced by H_2_O_2_/SD was significantly reduced by CB2 agonist (*P* < 0.05, Figure 6A, 6B). N-acetylcysteine (NAC) was selected for its common use as a negative control for ROS measurement. Furthermore, MDA, recognized as a marker for oxidative stress, is the product of oxidative free radical. Superoxide dismutase (SOD) and Glutathione (GSH) are important ROS scavengers which protect cells from ROS damage. As shown in Figure 6C-6E, CB2 agonist treatment significantly inhibited H_2_O_2_/SD-induced MDA up-regulation and restored H_2_O_2_/SD-reduced GSH level and SOD activity (*P* < 0.05, respectively).

### CB2 agonist enhanced the production of paracrine growth factors and decreased profibrotic cytokines including TGF-β1 and PDGF in AD-MSCs

Next, the effects of CB2 agonist treatment on the production of paracrine growth factors in AD-MSCs were also probed. Our data unveiled a significant enhancement of the production of paracrine growth factors including VEGF, bFGF, HGF, and IGF-1 and a markedly reduction of profibrotic cytokines including TGF-β1 and PDGF in CB2 agonist treated AD-MSCs compared with non-treated AD-MSCs (*p* < 0.05, Figure 7A-7F ).

### CB2 agonist activated Stat3 signaling in AD-MSCs subjected to H_2_O_2_/SD

**Figure 8:**
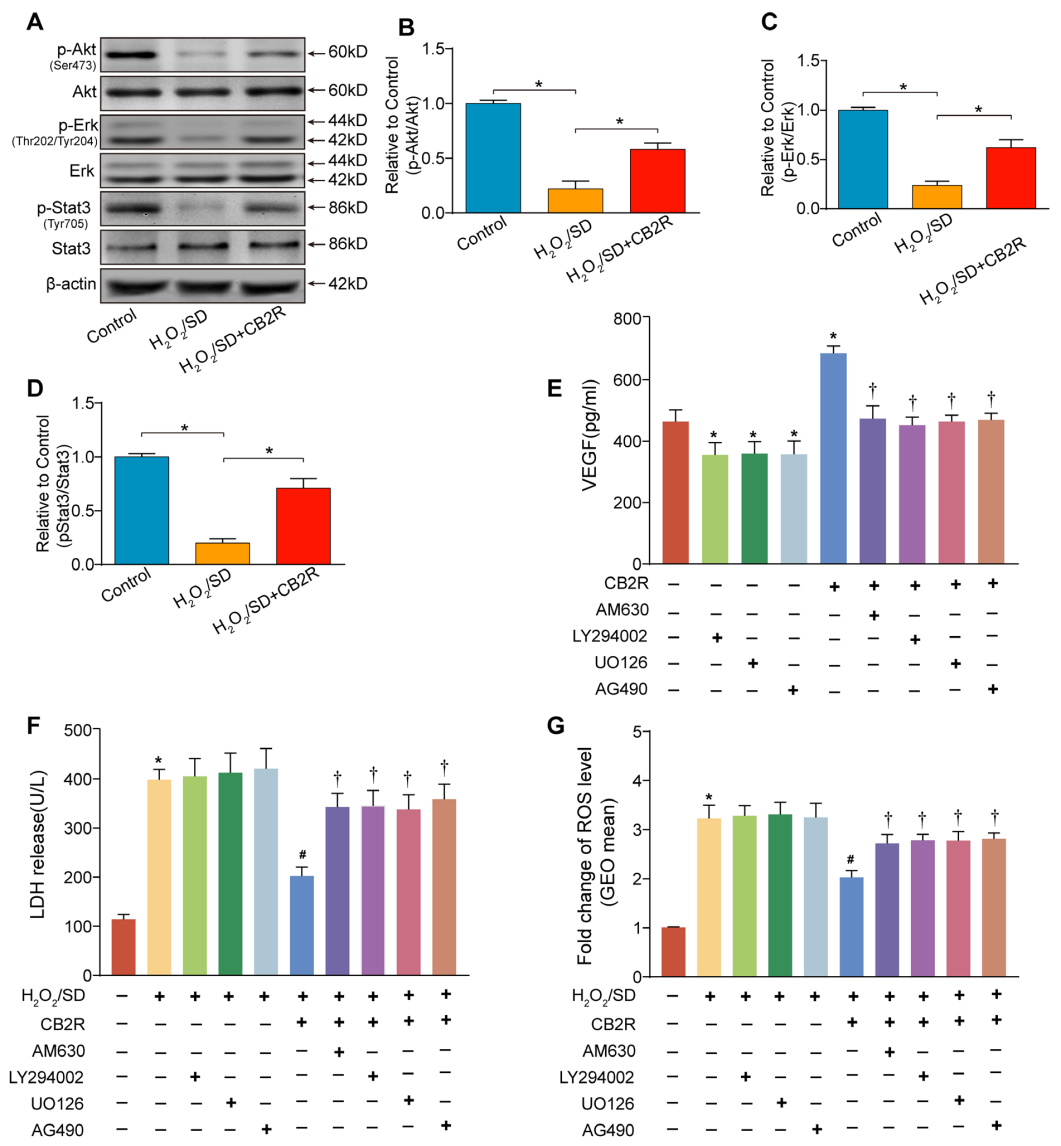
Activation of Stat3 through the phosphorylation of Akt and Erk1/2 was required for CB2 agonist-elicited beneficial actions in AD-MSCs **A.** Representative photographs of immune blot of Akt, Erk, Stat3 and their phosphorylation. (*n* = 3), **B.**-**D.** Quantitative analysis of **A.**. *P < 0.05 between indicated groups. AD-MSCs were pre-treated with CB2 agonist (5 μM, 12 h) in the presence or absence of AM630 (4μM, a CB2 inhibitor), U0126 (5μM, an Erk1/2 inhibitor), LY294002 (5μM, a PI3K/Akt inhibitor) or AG490 (a Stat3 inhibitor, 40μM) and subsequently underwent H_2_O_2_/SD insult. **E.** Paracrine growth factor VEGF secreted by AD-MSCs following indicated treatment, **F.** LDH release by AD-MSCs after indicated treatment, *n* = 3. **G.** Flow cytometry analysis of ROS production in AD-MSCs after indicated treatment, *n* = 3. *n* = 3. *P < 0.05 *versus* blank control, ^#^*P* < 0.05 *versus* H_2_O_2_/SD, ^†^*P* < 0.05 *versus* H_2_O_2_/SD+CB2R or CB2R.

As previous studies suggested that Stat3 activation mediated by the phosphorylation of Akt and Erk1/2 was involved in CB2-elicited cardio-protective and anti-oxidative effects [15], we next examined p-Akt(Ser473)/Akt, p-Erk(Thr202/Tyr204)/Erk, p-Stat3 (Tyr705)/Stat3 activity by Western blot to understand the possible mechanisms underlying CB2 agonist-conferred beneficial actions (Figure 8A-8D). As expected, CB2 agonist treatment markedly increased the levels of p-Akt(Ser473), p-Erk(Thr202/Tyr204), and p-Stat3 (Tyr705) in AD-MSCs subjected to H_2_O_2_/SD(*P* < 0.05). Taken together, these results indicated that the cyto-protective effect of CB2 agonist might be related to Stat3 activation than is mediated by the phosphorylation of Akt and Erk1/2.

### Activation of Stat3 through the phosphorylation of Akt and Erk1/2 was required for CB2 agonist-elicited beneficial actions in AD-MSCs

Next, to further elucidate the necessity of Stat3 signaling in CB2 agonist-elicited cyto-protection in AD-MSCs. AD-MSCs were pre-treated with CB2 agonist (5 μM, 12 h) in the presence or absence of AM630 (4μM, a CB2 inhibitor), U0126 (5μM, an Erk1/2 inhibitor), LY294002 (5μM, a PI3K/Akt inhibitor) or AG490 (a Stat3 inhibitor, 40μM), all from Selleck Chemicals (Houston, TX, USA). We chose the dose of AM630, LY294002, U0126 and AG490 based on previous reports with minor modifications [16, 17]. The chosen doses of four inhibitors have been verified to not only have significant inhibitory effects on respective targets, but also exhibit no significant toxic effects on cellular viability under normal condition. Intriguingly, the beneficial actions of CB2 agonist including depression of LDH release, attenuation of ROS level and up-regulation of VEGF secretion were abolished by the treatment with AM630, LY294002, U0126, or AG490(*P* < 0.05, Figure 8E-8G). Meantime, inhibitors only slightly but insignificantly increased the level of LDH release and ROS production in AD-MSCs exposed to H_2_O_2_/SD(*P* > 0.05 *vs.* blank control ), whereas inhibitors only remarkably decreased the production of VEGF in AD-MSCs cultured in normal condition(*P* < 0.05 *vs.* blank control). These findings verified the necessity of Stat3 signaling in CB2 agonist-elicited beneficial actions possibly through the activation of CB2 and subsequent phosphorylation of Akt and Erk1/2.

## DISCUSSION

In this study, we combined CB2 agonist treatment and AD-MSCs transplantation for treating MI injury in mice, and found that CB2 agonist improved AD-MSCs survival thereby enhancing its therapeutic effect after transplantation. Our data further showed that the pro-paracrine, anti-apoptotic and anti-oxidative function of CB2 agonist on AD-MSCs was associated with CB2 activation and subsequent Stat3 activation mediated by the Akt and Erk1/2. Since the good representative value of ischemic heart diseases in various ischemic diseases, combination of CB2 agonist treatment with stem cell therapy is of clinical significance to treat other ischemia associated disorders in a much broader context.

Previous studies have indicated the feasibility of CB2 activation in the optimization of stem cell based therapy for ischemic heart disease. On one hand, numerous experimental studies have clearly demonstrated the cardioprotective effect of CB2 activation [18]. Our previous research series also found that activation of CB2 in murine infarcted myocardium could activate endogenous cardiac repair and palliate myocardial fibrosis by improving the ischemic microenvironment [6, 7]. On the other hand, CB2 activation has been revealed to play a crucial part in stem cell physiology. Xie J and colleagues reported that CB2 activation could up-regulate the immunomodulatory effects of mouse bone-marrow derived mesenchymal stem cells [9]. In another report, CB2 activation reportedly enhanced osteogenic differentiation of bone marrow derived mesenchymal stem cells [8]. In line with these findings, our present study showed that CB2 activation enhanced the survival of transplanted AD-MSCs, as is evidenced by the increased BLI signals. Furthermore, combined treatment of CB2 agonist and AD-MSCs synergetically decreased cardiac fibrosis, improved cardiac function, inhibited cardiac apoptosis and attenuated myocardial oxidative stress. These findings suggest that the AD-MSCs transplantation adjuvant with CB2 agonist provided a promising strategy for facilitation of stem cell-based therapy for CHD. Worthy of note, we used intraperitoneal injection of AM1241 rather than MSCs pretreatment by AM1241 in current study, thus the *in vivo* effects of AM1241 might mainly be ascribed to the improvement of ischemic/oxidative microenvironment rather than the stem cell itself. By improving the ischemic microenvironment post MI, AM1241 not only favors a role in activation of endogenous myocardial regeneration as we previously demonstrated [7], but also promotes exogenous MSCs-mediated cardiac repair, depicting its multiple roles in facilitation of cardiac repair.

Recent evidences have highlighted the significance of controlling redox status in stem cell-based therapy [19]. Oxidative stress in ischemic heart alters the balance of the production and elimination of intracellular oxygen-free radicals and contributes to over-accumulation of reactive oxygen species (ROS), which are major factors inducing the death and loss of engrafted stem cells [20]. Besides, long-term survival of engrafted stem cells has been proved to be a prerequisite for stem cell therapy [21]. Furthermore, improving ischemic microenvironment by suppressing excessive oxidative stress had been demonstrated to improve efficacy of stem cells in ischemic injury [20]. Previously we have reported that melatonin and liver X receptor agonist, with their antioxidant properties, could facilitate AD-MSCs mediated cardiac repair [22, 23]. Similarly, our present study revealed that CB2 agonist with its anti-oxidative properties, could promote the survival of AD-MSCs, thereby enhancing their therapeutic potency. On the other hand, recent studies clearly show that enhancing antioxidant production at cellular levels enhances stem/progenitor cell functionalities, including proliferation, long-term survival in ischemic tissues, paracrine functions and complete differentiation of transplanted cells into mature cells [24]. In present study, by using H_2_O_2_/SD to mimic *in vivo* ischemic insult, we provided evidences that CB2 activation could enhance cellular antioxidants production, including SOD and GSH, thereby enhancing the viability and functionalities of AS-MSCs exposed to ischemic insult.

Our present study also observed the enhanced paracrine actions of AD-MSCs by CB2 agonist. To the best of our knowledge, this might be ascribed to CB2 agonist elicited Akt activation. MSCs overexpressing Akt (MSC-Akts) upregulate genes that encode for soluble factors, including VEGF, FGF-2, HGF, and insulin-like growth factor-I (IGF-I), which may be responsible for mediating cardioprotective effects [25]. In our previous research, inhibition of inositol hexakisphosphate kinases (IP6Ks), a physiologic inhibitor of Akt could activate Akt, thus enhancing the paracrine actions of MSCs [26]. In present study, we also observed the Akt activation en route to the enhanced paracrine actions of AD-MSCs by CB2 agonist, this effect could be abolished by Akt inhibitor LY294002. Collectively, these evidences highlighted the significant role of Akt signaling in paracrine actions MSCs. Besides, Stat3 activation might also account for the enhanced paracrine effects of AD-MSCs by CB2 activation. Previous studies have unveiled that inhibition of Stat3 signaling could abolish the paracrine actions of MSCs [13]. Our current study achieved a similar result, further stressing the significance of Stat3 signaling in MSCs’ paracrine actions. In addition, our result depicted that profibrotic cytokines such as TGF-β1 and PDGF were inhibited by CB2 agonist in AD-MSCs, facilitating the interpretation of the anti-fibrotic effects of CB2 agonist in infarcted hearts.

Our present study further depicted a role of Stat3 activation en route to the beneficial effects of CB2 agonist. Stat3 is strongly involved in many cellular processes including cell apoptosis, autophagy, inflammation, immune response, oxidative stress, and angiogenesis [12]. Studies have also demonstrated that kinases, such as Akt and Erk1/2, could phosphorylate Stat3 [27, 28]. Besides, it is well established that the signalling pathways Akt, Erk and Stat3 are powerful intrinsic pro-survival signaling cascades to promote cell survival in stress conditions. Liu JF and colleagues previously reported that Exendin-4 could improve the survival and therapeutic efficacy of engrafted AD-MSCs through Stat3 activation *via* the phosphorylation of Akt and Erk1/2 [13]. Consistently, our results demonstrated that Stat3 could be activated by CB2 agonist through the phosphorylation of Akt and ERK1/2 both in AD-MSCs and ischemic myocardium. Previous report have revealed a direct link between Stat3 signaling and CB2 activation in the cardio-protection of ischemia/reperfusion injury, which is consistent with our findings [15]. Furthermore, by using specific inhibitors, we provided evidences that activation of Stat3 through the phosphorylation of Akt and Erk1/2 was required for CB2 agonist-elicited beneficial actions in AD-MSCs. This is the first attempt to elucidate the association between Stat3 signal and CB2 agonist-mediated cyto-protective properties in AD-MSCs.

Despite the clinical relevance, our current study suffered from certain limitations. First, although the utilization of chemical inhibitors could to some extent verify the necessity of respective signaling molecules, gene silencing methods might be more convincing. Second, we did not perform GFP staining to detect engrafted MSCs in cardiac tissues, albeit Fluc activity of MSCs has been examined in cardiac tissues.

In conclusion, our current work demonstrated a favorable role of CB2 agonist in stem cell based therapy for experimental myocardial infarction. CB2 agonist could effectively enhance the survival and viability of AD-MSCs engrafted in ischemic myocardium. Combined treatment of CB2 agonist and AD-MSCs has a synergistic effect on restoration of cardiac functions. Besides, CB2 agonist could protect AD-MSCs against H_2_O_2_/SD-induced injury through Stat3 activation *via* the phosphorylation of Akt and ERK1/2. Our data support the promise of CB2 agonist as a novel strategy to improve MSC-based therapy for ischemic diseases.

## MATERIALS AND METHODS

### Experimental animals

AD-MSCs were isolated from firefly luciferase (Fluc) and enhanced green fluorescence protein (eGFP) double positive transgenic mice (Tg [Fluc-egfp]) mice (FVB/N background) as we previously described [29, 30]. Syngeneic female FVB mice with the same genetic background as Fluc^+^-eGFP^+^ transgenic mice (8 weeks old, 20-25g) was subjected LAD ligation for the myocardial infarction (MI) model and served as hosts for transplanted AD-MSCs. This setting of cell recipients and cell donors should greatly minimize the immunogenicity raised by allogeneic MSCs. All procedures were performed in accordance with the National Institutes of Health Guidelines on the Use of Laboratory Animal. Experimental protocols and animal care methods were approved by the Fourth Military Medical University Committee on Animal Health Care.

### Animal grouping and treatment

FVB mice (*n* = 100) were divided into 5 groups: (1) Sham group(*n* = 20); (2) MI+ PBS group (PBS, *n* = 20); (3) MI+AD-MSCs group (AD-MSCs, *n* = 20); (4) MI+ CB2 agonist AM1241 group (CB2R, *n* = 20); (5) MI+ AD-MSCs +AM1241 group (AD-MSC+CB2R, *n* = 20). MI was accomplished by ligation of the left anterior descending (LAD) as we described before [22]. 30 minutes after the construction of MI model, cell suspensions or PBS were directly injected into the ischemic border zone of the myocardium at four different sites (5μl to each site) with a total volume of 20 μL containing 1x10^6^ cells with the help a Hamilton syringe with a 29-gauge needle. AM1241 (Selleck, USA, 20mg/kg/d) was intraperitoneal injected into mice for successive 28 days after cell transplantation in mice of group (4) and (5). AM1241 was dissolved in vehicle (18:1:1 ratio of normal saline: emulphor: ethanol).

### Isolation, cultivation and identification of AD-MSCs

The isolation of AD-MSCs were performed in accordance with a modified procedures that we described previously [30]. Immuno-phenotype and multilineage differentiation of cultured AD-MSCs were identified as we previously described [30, 31].

### BLI for AD-MSCs imaging

*In vivo* Bioluminescence imaging (BLI) was employed to track the survival and retention of engrafted AD-MSCs. After the anaesthesia and intra-peritoneal injection of 150 mg/kg D-luciferin (Invitrogen, USA), Mice were imaged by IVIS Xenogen Kinetic system (Caliper Life Sciences, USA) as described previously [30]. Fixed-area region of interests (ROIs) were created over murine heart, and photons emitted from the ROIs were quantified by P s^-1^cm^-2^sr^-1^ using Living Image software (Caliper, MA, USA). For *in vitro* imaging, cells after respective treatment were incubated with D-luciferin reporter probe (2.25 ng/μl, Invitrogen), and then measured using the IVIS Xenogen Kinetic system.

### *Ex vivo* luciferase assay

To detect luciferase activity in engrafted AD-MSCs, heart tissues were removed from sacrificed mice on POD14, homogenized in PBS containing a protease inhibitor cocktail (Selleck, USA), and lysed with 1×PLB(passive lysis buffer). Luciferase activity in the supernatant was measured using the Luciferase Assay System (Beyotime, China) on GloMaxTM 20/20 Luminometer (Promega, USA) following the protocols we previously described [22].

### Left ventricular functional analysis with echocardiography

Echocardiography for non-invasive evaluation of left ventricular function were performed with VEVO2100 ultrasound system (VisualSonics, Canada) as we have previously described [31]. Briefly, mice were anesthetized with inhaled 2% isoflurane, two-dimensional and M-mode images were recorded using a 30-MHz linear array ultrasound transducer. All measurements were based on 3 consecutive cardiac cycles. Left ventricular ejection fraction (EF) and fractional shortening (FS), two main indicator of left ventricular function, were calculated by computer algorithms.

### Histological staining

The myocardium was fixed in 4% paraformaldehyde and sectioned at 4-5 µm. Myocardial fibrosis were observed by Masson’s trichrome staining to indicate infarction area within the left ventricle (LV). Myocardial apoptosis was evaluated by a terminal deoxynucleotidyl transferase dUTP nick-end labeling (TUNEL) assay kit(Roche, Nutley, NJ, USA), following the manufacturer’s instructions. Myocardial reactive oxygen species generation was examined by csonfocal microscope *via* in-situ DHE staining as we previously described. The immunoreactive areas were analyzed with Image-Pro Plus 4.5 software (Media Cybernetics, Silver Spring, USA).

### H_2_O_2_/SD injury to simulate MI

The H_2_O_2_/serum deprivation injury cell model was used to mimic MI as we have previously described [13]. In brief, after indicated treatment, AD-MSCs were exposed to hypoxia (94% N2 , 5% CO2 and 1% O2 ) in an anaerobic system (Thermo Forma, USA) at 37 °C for 12 hours in Hanks buffer supplemented with 100 μM H_2_O_2_.

### Flow cytometry for ROS production

The sensitive ROS fluorescent probe-DCFH-DA (KGT010-1; KeyGEN BioTECH, Nanjing, China) was used to detect the ROS accumulation following the manufacturer’s instructions. In brief, AD-MSCs were incubated with 5 μM DCFH-DA at 37°C for 30 min. The fluorescence was then detected on flow cytometer (Becton Dickinson Biosciences, Franklin Lakes, NJ).

### Biochemical analyses of malondialdehyde (MDA), glutathione(GSH), LDH release, caspase-3 activity, superoxide dismutase(SOD) activity and paracrine factors

The level of MDA, GSH, SOD activity, LDH release, caspase-3 activity and paracrine factors including VEGF, HGF, bFGF, TGF-β, PDGF and IGF-1 in the supernatant of AD-MSCs or cardiac tissues were measured by commercially available assay kit (Nanjing Jiancheng Bioengineering Institute, China) in accordance with the manufacturer’s protocols. All samples were stored at -80°C before assay and the concentration data in the samples were then measured by comparing the OD450 to the standard curves.

### Measurement of the levels of O_2_−

O_2_^−^ production in cardiac tissues was assayed using lucigenin chemiluminescence method [32]. Briefly, cardiac tissues were rinsed and homogenized. The supernatant samples were loaded with dark-adapted lucigenin (5 μM), then read in 96-well microplates using a luminometer (GloMax, Promega). Light emission was recorded for 5 min and was normalized to tissue dry weights.

### Western blot analysis

Myocardium tissues and AD-MSCs were harvested for Western Blot following standard protocol. Samples consisting of 50 μg total protein were loaded onto an SDS-PAGE gel (Beyotime, China) and transferred electrophoretically to nitrocellulose membranes (LC2000, Invitrogen, USA). After blocked with 5% bovine serum albumin in PBS, the membranes were incubated with the appropriate primary antibody against Akt, phospho-Akt, ERK, phosphor-Erk, Stat3, phosphor- Stat3, all from Cell Signaling Technology, Danvers, MA,USA) overnight. The next day, the blots were washed and incubated in the appropriate secondary antibodies (Abcam, Cambridge, MA, USA) at room temperature for 1 h. Immunoreactivity was then detected by sequential incubation with HRP-conjugated antibodies and enzymatic chemiluminescence. Quantitative analysis was performed using QuantiOne imaging software (Bio-Rad, USA) to assess the integrated optical density (IOD) of each band.

### Statistical analyses

All values were presented as means ± standard error. All statistical tests were performed using Graphrad Prism software version 6.02 (Graphpad Software, CA, USA). Comparison between groups was subjected to analysis of ANOVA followed by Bonferroni correction for post hoc *t*-test. Two-sided tests have been used and P value less than 0.05 were considered statistically significant.

## Abbreviations

CB2: cannabinoid receptor type II
AD-MSCs: adipose-derived mesenchymal stem cells
MI: myocardial infarction
Stat3: signal transducers and activators of transcription 3
TGF-β: transforming growth factor-β
PDGF: Platelet derived growth factor
H_2_O_2_/SD: hydrogen peroxide/serum deprivation
BLI: bioluminescence imaging
TUNEL: terminal deoxynucleotidyl transferase dUTP nick-end labeling assay
MDA: malondialdehyde
GSH: glutathione
SOD: superoxide dismutase
EF: left ventricular ejection fraction
FS: left ventricular fractional shortening
ROS: reactive oxygen species
